# Post-intervention sustainability of time-restricted eating versus caloric restriction: a secondary analysis

**DOI:** 10.1038/s41366-025-01968-2

**Published:** 2025-11-28

**Authors:** De-An Chen, Raul Herrera Pena, Niki Oldenburg, Qi Wang, Erika Helgeson, Brad Yentzer, Abdisa Taddese, Nicole LaPage, Emily N. C. Manoogian, Satchinananda Panda, Lisa S. Chow

**Affiliations:** 1 Department of Medicine, University of Minnesota, Minneapolis, MN USA; 2 Clinical and Translational Science Institute, University of Minnesota, Minneapolis, MN USA; 3 School of Public Health, University of Minnesota, Minneapolis, MN USA; 4 Salk Institute for Biological Sciences, San Diego, CA USA

**Keywords:** Obesity, Weight management

## Abstract

Rising obesity rates necessitate sustainable weight management strategies. Current lifestyle guidelines focus on reducing caloric intake through personalized interventions to promote compliance. This secondary analysis evaluated post-intervention sustainability of time-restricted eating (TRE) versus caloric restriction (CR), hypothesizing that TRE’s “watching the clock” approach may be more sustainable than CR’s “watching calories.” Following a 12-week supervised intervention (TRE: 8-h eating window, *n* = 29; CR: 15% caloric reduction, *n* = 26), 41 participants (75%; 24 F/17 M; 23 TRE/18 CR; age 43.1 ± 11.6 years; BMI 34.7 ± 5.4 kg/m²) completed follow-up surveys at 1, 3, and 6 months. TRE participants maintained weight across all follow-ups compared to final intervention weight. CR participants showed significant loss at 1 month (−1.6 ± 2.5 kg, *p* = 0.02), returning to baseline by 3 months. Both interventions had similar continuation rates (1,3,6 months: TRE: 52%, 36%, 47%; CR: 63%, 57%, 50%; *p* = 0.60) and recommendation rates (TRE: 81%, 85%, 86%; CR: 88%, 86%, 80%; *p* = 0.72). TRE participants reported improved sleep, energy, and digestion but experienced morning hunger and scheduling challenges. CR participants noted increased food mindfulness but reported tracking anxiety, cravings, and potential binge eating. Despite limitations including small sample size and self-reported weight, both self-sustained TRE and CR showed similar acceptability and weight maintenance at 3–6 months post-intervention. Clinical Trial Registration: Clinicaltrials.gov NCT04259632.

Obesity presents a health challenge, with US adult obesity prevalence rising from ~38% (2013–2014) to ~41% (2021–2023) and severe obesity (BMI ≥ 40 kg/m^2^) increasing from ~7 to ~10% [1]. The 2025 American Diabetes Association (ADA) guidelines for “Obesity and Weight Management for the Prevention and Treatment of Type 2 Diabetes” recommends reducing caloric intake to target 3–7% weight loss, with >10% weight loss having greater benefits. These interventions include high-frequency counseling ( ≥16 sessions in 6 months) focusing on nutrition, physical activity, and behavioral strategies to achieve a 500–750 kcal/day deficit [2]. The ADA recommends individualizing nutritional approaches rather than prescribing a specific plan or macronutrient composition [3]. While time-restricted eating (TRE) is not considered a traditional weight loss intervention, TRE trials have demonstrated ~5% weight loss in people with obesity without diabetes [1, 4–6].

Caloric restriction (CR) attrition rates commonly exceed 20% [7], attributed to extrapersonal challenges (environmental/social situations) and intrapersonal challenges (self-blame and habit maintenance) [8]. In contrast, TRE limits daily eating to 6–10 h without calorie restriction [9]. TRE’s appeal lies in its simplicity—“watching the clock” versus “watching the calories”—potentially offering greater sustainability. Both approaches reduce weight in supervised settings [5, 6, 10], but post-intervention continuation and the sustainability of weight loss remain unknown. This knowledge gap is critical since long-term weight loss depends on intervention sustainability.

This secondary analysis examines self-reported adherence to TRE or CR at 1, 3, and 6 months following a 12-week supervised intervention [6]. We hypothesized that TRE would demonstrate greater sustainability than CR due to its simpler implementation. Additionally, we sought to understand participants’ qualitative experiences to inform future TRE/CR studies.

## Study design

This secondary analysis examined follow-up data after a 12-week randomized controlled trial comparing TRE, CR, and unrestricted eating (UE) for weight loss [6]. Participants with obesity (BMI ≥ 30 kg/m²) were recruited from MHealth Fairview Health System, Minnesota (10/2020-10/2023). The study received University of Minnesota IRB approval (STUDY00008545), was registered at ClinicalTrials.gov (NCT04259632), and all participants provided written informed consent. The primary study’s main outcome was weight change from baseline to 12 weeks. The protocol and statistical analysis plan are in the supplemental material of the original publication [6].

**Fig. 1:**
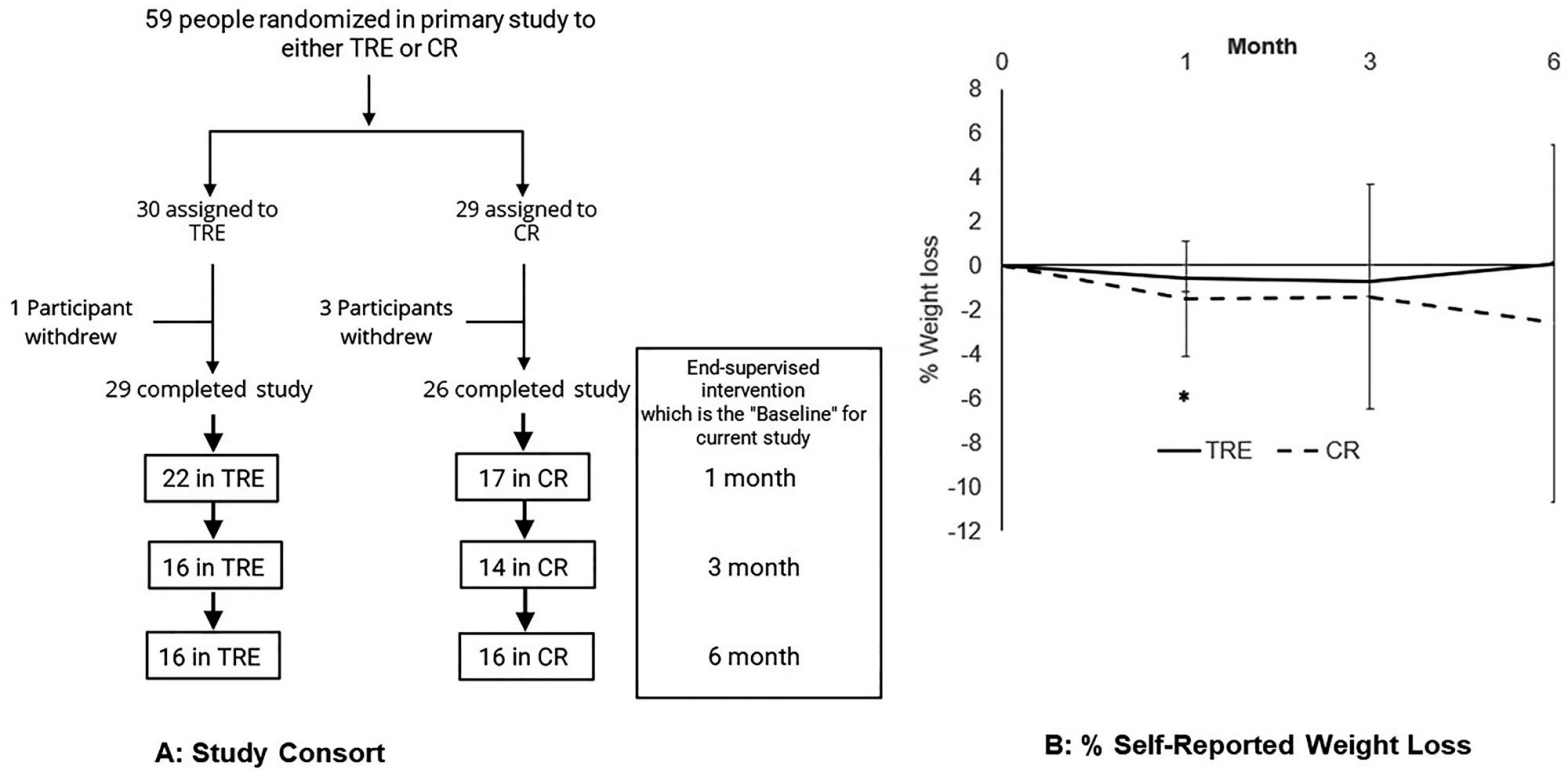
Study Flow and Reported Weight Loss. **A** Study Consort Diagram. *A total of 41 participants completed at least 1 survey. Two participants did not complete a survey at 1 month but completed a subsequent survey. **B** % Self-Reported Weight Loss * Indicates significant weight loss relative to baseline in the CR group. Respondents at Time Points (TRE/CR): Month 1 (20/15), Month 3 (15/13), Month 6 (15/14). % Weight change for TRE [mean(SD)] : (Month 1: −0.5% (1.7), Month 3: −0.7% (2.6) Month 6: −0.1% (3.2) % Weight change for CR [mean(SD)] : (Month 1: −1.5% (2.6), Month 3: −1.4% (5.1) Month 6: −2.6% (8.1).

For this secondary analysis, only the TRE and CR participants were analyzed. After completing the primary study, participants could continue, modify, or switch interventions. REDCAP surveys collected self-reported weight, continuation, dietary changes, benefits/challenges, recommendation likelihood, and comments at 1, 3, and 6 months post-intervention. Week 12 weight served as baseline (Fig. 1A). The primary endpoint was weight change at one-month post-intervention. Secondary endpoints included weight change at 3 and 6 months, continuation rates, recommendation rates, and intervention experience.

## Data analysis

For quantitative data, we used linear and binomial mixed effects models with subject-specific random intercepts to compare TRE and CR groups. Statistical significance was p < 0.05.

For qualitative data, we categorized responses as positive, mixed, or negative across intervention logistics, physical effects, and psychological/emotional effects. Two reviewers (LC, DC) analyzed free text responses using MAXQDA (VERBI Software, Berlin Germany). After anonymizing the data, reviewers developed a coding framework combining inductive and deductive approaches, coded all responses independently, identified responses with similar themes, and selected representative quotes (Table 1).

**Table 1:** Representative survey comments regarding the pros and cons of TRE/CR.

PROS	
TRE	CR
Making this a habit for three months has been great. It is so easy for me to wait to eat in the morning.	I did not feel hungry. It made me much more aware of what I was eating. I have made better food choices since leaving the study. I changed my mentality around how much food I really need per meal.
After the study, I experimented with different eating styles for a few weeks. Even trying calorie counting. I came back to TRE as it’s more sustainable for me.	I feel better and pay more attention to what kinds of foods I am eating.
**CONS**	
**TRE**	**CR**
Hard time concentrating in the late morning since I’m so hungry.	It increases some of my anxiety, and I tend to get hyperfocused on all of it - tracking, numbers, counting, timing, etc. While it may be physically healthy for me, I have not found it to be completely emotionally or psychologically healthy for me.
Just the difficulty with scheduling meals at times different than family.	I didn’t find a benefit… I binged after restricting.

## Participant characteristics

The primary study randomized 88 participants to TRE (8-hour eating window; *n* = 30), CR (15% caloric reduction; *n* = 29), or UE (*n* = 29). As published, TRE and CR achieved similar weight loss (TRE: −3.0 kg [95% CI: −4.8, −1.3]; CR: −4.1 kg [95% CI: −6.0, −2.2]; *p* = 0.69). Of 88 enrolled, 81 (92%) completed the study [mean ± SD: age 43.2 ± 10.5 years, BMI 36.2 ± 5.1 kg/m²; 54.5% female, 84.1% white] [6].

This secondary analysis includes 41 participants from TRE (*n* = 23) and CR (*n* = 18) groups who completed ≥1 follow-up survey. Participants were 59% female, 83% white, with mean age 43.1 ± 11.6 years, BMI 34.7 ± 5.4 kg/m², weight 103.2 ± 18.1 kg, fasting glucose 100.2 ± 10.3 mg/dL, HbA1c 5.5 ± 0.3%, total cholesterol 185.7 ± 34.2 mg/dL, LDL 110.5 ± 31.9 mg/dL, HDL 48.1 ± 11.0 mg/dL, and triglycerides 135.5 ± 72.6 mg/dL. From the post-intervention survey data, the TRE group reported an average eating window of 10:30—19:00 at 1 month (8.5 h), 12:00–20:00 at 3 months (8 h), and 10:45–19:00 at 6 months (8.25 h).

## Weight change

TRE participants maintained post-intervention weight at 1, 3, and 6 months. CR participants reported significant weight loss at 1 month (−1.6 ± 2.5 kg, *p* = 0.02), but not at 3 or 6 months. Percentage weight change illustrates these patterns (Fig. 1B). TRE weights remained stable with a slight, non-significant trend toward loss by 6 months ( ~ 3% decrease from baseline). CR participants showed significant weight loss at 1 month ( ~ 2% decrease, *p* = 0.02) with a non-significant trend at 6 months ( ~ 6% decrease).

## Intervention continuation

Both interventions showed similar continuation rates across follow-up (1, 3, 6 months: TRE: 52%, 36%, 47%; CR: 63%, 57%, 50%), with no significant between-group difference (*p* = 0.60). By 6 months, continuation rates were nearly identical ( ~ 47–50%).

## Recommendation likelihood

Recommendation rates to friends/family were comparable (1, 3, 6 months: TRE: 81%, 85%, 86%; CR: 88%, 86%, 80%; *p* = 0.72), suggesting value beyond personal adherence.

## Qualitative feedback

Common themes across both groups included improved weight loss, energy, and overall health.TRE participants reported reduced snacking and ease following structured eating windows while struggling with morning hunger affecting concentration and coordinating their eating window with work, family, and social events. Some TRE participants reported fewer digestion issues, while others noted weight gain—an effect not reported by CR participants. CR participants noted increased mindfulness about food choices and portions while finding tracking intake and meal preparation burdensome. CR participants reported benefiting from increased awareness of eating habits and understanding their behaviors while reporting anxiety about tracking intake, perceived food restrictions, and compliance burden sometimes triggering post-restriction binge eating (Table 1).

## Discussion

This secondary analysis evaluated post-intervention sustainability of TRE and CR following a 12-week supervised intervention, hypothesizing TRE would be more sustainable due to its “watching the clock” versus “watching the calories” approach. The CR group reported significant weight loss at 1-month post-intervention, while TRE did not. At 6 months, both groups maintained their post-intervention weight loss with similar continuation and recommendation rates, though each faced distinct adherence challenges. Both interventions demonstrated high acceptability and weight maintenance over 6 months of follow-up.

Several studies, including ours [5, 6], show CR and TRE produce similar weight loss ( ~ 3–5 kg) in patients with obesity, but their sustainability outside a structured intervention remains unclear. This research examines the durability of voluntary TRE/CR continuation. At 6 months post-intervention, approximately half of respondents maintained their assigned intervention. Both groups highly recommended their approaches despite varied adherence, suggesting perceived value beyond personal continuation. Each intervention showed distinct patterns: TRE participants reported improved sleep, energy, digestion, and appreciated its simplicity; CR participants noted greater food awareness but experienced tracking anxiety and potential post-restriction binge eating.

This study directly compares voluntary continuation of TRE versus CR after a structured clinical trial. Previous TRE sustainability research examined TRE in isolation rather than compared to CR. One study of 20 participants after a 3-month TRE intervention (10-h eating window) found only seven maintained strict windows while ten adopted modified approaches [11, 12]. Another study surveyed participants ~16 months post-intervention, finding 26.3% maintained original TRE, 36.8% practiced modified TRE, and 36.8% discontinued completely [13]. A scoping review concluded that sustained TRE maintenance requires individualized adjustments and support [14]. This study’s main finding is that both TRE and CR groups maintained post-intervention weight during unsupervised follow-up with high intervention acceptability.

CR’s rigor during supervised intervention may explain significant 1-month post-intervention weight loss, possibly reflecting selection bias toward compliant participants. However, similar weight loss between groups by 3 months highlights CR’s sustainability challenges and suggests both interventions are viable for weight maintenance. Given TRE’s simplicity, future research should examine its long-term efficacy in high metabolic risk populations, such as augmenting GLP-1/GIP agonist weight loss, minimizing medication dosing while maintaining efficacy, or implementing TRE around bariatric surgery to maximize outcomes.

Clinical implications include tailored approaches based on individual preferences, as continuation rates were similar despite different qualitative experiences. The interventions may work synergistically, though evidence is mixed [4, 15]. For TRE, addressing morning hunger and social eating challenges could improve adherence. For CR, simplified tracking and reduced psychological distress around restriction would be beneficial. Emphasizing non-weight benefits (improved sleep, energy, mindfulness) may enhance long-term motivation.

A strength of this study is the follow-up of randomized TRE and CR participants, enabling direct comparison between groups who voluntarily continued their assigned interventions. However, several limitations from the original study and this secondary analysis could impact results, including small sample size, self-reported adherence and weight change, and a relatively healthy population (other than obesity) without diabetes. Analyses were restricted to survey respondents and may not represent all participants’ experiences.

## Conclusion

Both TRE and CR groups showed moderate continuation rates, high recommendation rates, and distinct benefit/challenge patterns after supervised intervention. These findings require cautious interpretation due to small sample size, self-reported measures, and relatively healthy population. At 3- and 6-month follow-up, both groups maintained weight achieved during the primary study. These findings highlight the importance of weight maintenance after structured interventions and demonstrate TRE/CR acceptability in supporting sustainability.

### Data sharing

**Protocol:** posted as data supplement with primary paper, which was published in Obesity [6].

**Documented analytic dataset:** Data available 1 year post-publication with IRB approval and MTAs, upon request to Dr. Chow’s team. This data will be provided de-identified.

**Statistical Code**: Upon request, the code available with the dataset to facilitate interpretation.

## Data Availability

By request from Dr. Chow.
